# Patients aged in their 70s do not have a high risk of progressive osteoarthritis following arthroscopic femoroacetabular impingement correction and labral preservation surgery

**DOI:** 10.1007/s00167-019-05520-4

**Published:** 2019-05-07

**Authors:** Eisaburo Honda, Hajime Utsunomiya, Akihisa Hatakeyama, Hirotaka Nakashima, Hitoshi Suzuki, Dean K. Matsuda, Akinori Sakai, Soshi Uchida

**Affiliations:** 1 Department of Orthopaedic Surgery, Wakamatsu Hospital of the University of Occupational and Environmental Health, 1-17-1, Hamamachi, Wakamatsu, Kitakyushu, Fukuoka 808-0024 Japan; 2 Department of Orthopaedic Surgery, Faculty of Medicine, University of Occupational and Environmental Health, 1-1, Iseigaoka, Yahatanishi, Kitakyushu, Fukuoka, 807-8555 Japan; 3 DISC sports and Spine, Marina Del Rey, USA

**Keywords:** Hip arthroscopy, 70 years old, Femoroacetabular impingement, Labral preservation

## Abstract

**Purpose:**

The purposes of this study were to (1) evaluate the effect of age on clinical outcomes of arthroscopic femoroacetabular impingement (FAI) with labral preservation surgery and (2) identify predictors of poor postoperative clinical outcomes.

**Methods:**

Eighty-four patients who underwent hip arthroscopic treatment for FAI between 2009 and 2013 were retrospectively reviewed. Patients were divided into three groups based on age. The Advanced age group consisted of patients over 70 years old, the Middle age group consisted of patients in their 50s and 60s, and the Younger age group consisted of patients less than 50 years of age. Total hip arthroplasty (THA) conversion, radiographic progression of osteoarthritis and patient-reported outcomes including modified Harris Hip Score (MHHS) and Non-arthritic Hip Score (NAHS) were investigated.

**Results:**

The mean follow-up period was 32.2 (range 24–60) months. THA was required in 3 patients in their 50s and 60s, which was a significantly higher rate compared to that in patients Younger than 50 years old (17% vs 0%, *p* = 0.036). Progression to osteoarthritis was also significantly more frequent in patients in their 50s and 60s than in patients in their 70s (50s and 60s: 33%; 70s: 0%, *p* = 0.030). In all age groups, the preoperative MHHS and NAHS improved at last follow-up (*p* < 0.001). The 50s and 60s age group [hazard ratio (HR) 6.62], preoperative mild osteoarthritic change (Tönnis grade 1, HR: 3.29) and severe cartilage damage on the acetabulum (HR: 2.63) were risk factors for progressive osteoarthritis and THA conversion.

**Conclusions:**

Arthroscopic FAI correction and labral preservation surgery provide favourable clinical outcomes for patients over 70 years old in the absence of significant osteoarthritis and severe acetabular chondral damage. Patients in their 50s and 60s have a higher risk of both THA conversion and progressive osteoarthritis, while patients aged over 70 years show no evidence of progressive osteoarthritis. Chronologic age in isolation is not an absolute contra-indication to hip arthroscopy.

**Level of evidence:**

III.

## Introduction

Femoroacetabular impingement (FAI) is a condition resulting from the pathologic abutment between the acetabular rim and proximal femur [10]. It has been recognised that FAI may cause labral tears and/or chondrolabral damage with a predisposition toward osteoarthritis in recent Level II and III studies [1, 10, 26, 31, 39]. Arthroscopic surgery for FAI in the younger population is a safe and effective procedure, especially in patients younger than 50 years old [4, 39].

In contrast, several studies have shown less favorable clinical outcomes following arthroscopic surgeries for treating FAI patients in the older population. Javed et al. demonstrated, in a series of 40 patients over 60 years old, that seven of 40 (17%) patients required total hip arthroplasty (THA) [15]. Domb et al. reported in a series of 52 patients over 50 years old that 17.3% underwent subsequent THA [9]. Philippon et al. showed that 20–32% of patients 50 years old or older required conversion to THA [28, 30].

Previous studies have reported that the presence of osteoarthritic changes at the time of hip arthroscopic surgery for FAI patients negatively affects postoperative clinical outcomes [6]. Byrd et al. showed that 52 patients who underwent hip arthroscopy with 10 years of follow-up had a 26.9% THA conversion rate, and the presence of arthritis at the time of index procedure was an indicator of a poorer prognosis [5]. Sansone et al. reported that arthroscopic treatment for FAI with OA (Tönnis grade 1 or 2) resulted in improvement of postoperative PROs and a 7% THA conversion rate [33]. Moreover, Philippon et al. concluded in their analysis of patients older than 50 years that the most important predictors of THA were patients who had less than 2 mm of joint space [28].

Other factors have been associated with poor clinical outcomes after surgery, including preoperative PROs (higher Western Ontario and McMaster Universities Osteoarthritis Index and lower MHHS), patients’ characteristics (higher body mass index and workers’ compensation), and arthroscopic procedure and morphology (radiographic acetabular coverage and offset in the superior portion of the femoral neck) [8, 12, 22, 29].

In the older population, there exists a knowledge gap of risk factors for poor clinical outcomes including conversion to THA following arthroscopic treatment of FAI patients with preserved joint space (greater than 2 mm). The purposes of this study were (1) to investigate the effect of age on clinical outcome of arthroscopic FAI with labral preservation surgery and (2) to identify predictors of poor postoperative clinical outcomes. It was hypothesised that arthroscopic FAI and labral preservation surgery could provide favorable clinical outcomes for treating patients of all ages, but older patients ≥ age 50 years would have poorer clinical outcomes and higher THA conversion rates. This study may provide clinical relevance if there is a subset of older patients who may do particularly well following hip arthroscopy for FAI.

## Materials and methods

Approval for the study was granted through the institutional review board IRB (Authorization number H29-004). Records of 318 patients who underwent hip arthroscopic treatment by a single surgeon (senior author S.U.) were retrospectively reviewed in our institution (Wakamatsu hospital of University of Occupational and Environmental Health) between 2009 and 2013. Clinical inclusion criteria were groin pain refractory to a minimum of 3 months of nonoperative treatment, which included activity modification, physical therapy, and non-steroid anti-inflammatory agents, and physical findings of restricted hip range of motion (ROM) (flexion < 105° and/or restricted internal rotation in flexion < 20°) and a positive impingement test. All patients underwent a diagnostic intra-articular injection of local anaesthetic with temporary pain relief [16, 40]. The anterior impingement test (flexion adduction internal rotation, FADIR) was performed with the patient in the supine position with an internal hip rotation at 90° flexion and 10° adduction [35]. Radiographic evidence of a cam deformity included an alpha angle > 55° and/or head–neck offset ratio < 0.14 on at least one radiographic and/or computed tomography (CT) or magnetic resonance imaging (MRI) image [7]. Radiographic evidence of a pincer deformity was defined as a positive crossover sign in the presence of a lateral centre edge (LCE) angle ≥ 30°, an LCE angle of ≥ 40° and/or an acetabular inclination of < 0° [19]. Radiographic FAI subtype was additionally classified as isolated cam, isolated pincer, or combined FAI. Intra-articular pathological abnormalities including acetabular labral and chondral lesions were evaluated by gadolinium-enhanced 1.5-T magnetic resonance (MR) arthrography or 3-T MRI. Patients with a minimum postoperative follow-up of 2 years were included (mean, 32.2 ± 13.6 months range 24–60 months). Exclusion criteria included patients with developmental dysplasia of the hip (DDH) (LCE angle < 25°, 79 hips), osteoarthritis (OA) with Tönnis grade ≥ 2 and/or joint space < 2 mm (20 hips) [34], DDH and OA (12 hips), synovial osteochondromatosis (6 hips), previous pelvic trauma (6 hips), previous hip surgery (4 hips), Legg–Calve–Perthes disease (2 hips) or other disorders (15 hips). Seventy-six patients (*n* = 152) with bilateral symptomatic hip conditions were excluded.

**Fig. 1:**
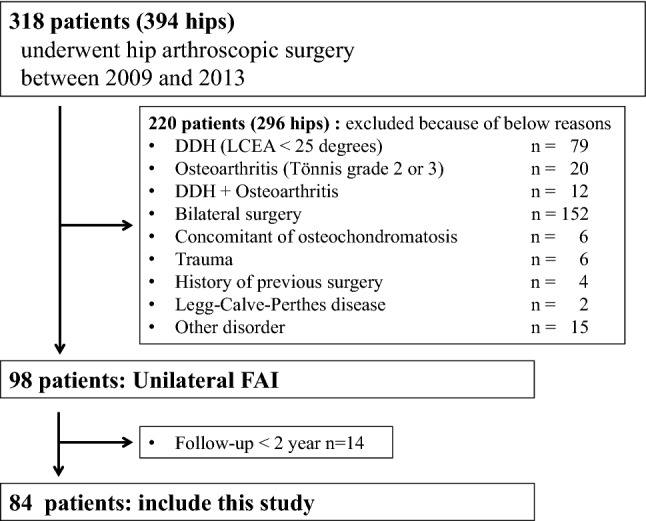
Flow diagram showing patient inclusion criteria for this study

Fourteen patients (14 hips) were lost to follow-up, with the resultant 84 patients (84 hips, 85.7% follow-up rate) comprising the focus of this study (Fig. 1). The mean age at the time of surgery was 41.0 (range 13–78) years with 43 male and 41 female patients.

### Subject grouping

Patients were divided into three groups by age: the Advanced age group consisted of 9 patients (6 males and 3 females) over 70 years of age (median 73 years, range 71–78) at the time of surgery, the Middle age group consisted of 18 patients (6 males and 12 females) aged from 50 to 69 years (median 57 years, range 51–63), and the Younger age group consisted of 57 patients (31 males and 26 females) less than 50 years of age (median 30, range 13–49 years).

### Arthroscopic evaluation

Initial arthroscopic diagnostic evaluation of the labral condition and chondral lesions of the acetabular rim and femoral head were documented. The cartilage condition of the acetabular rim lesion was evaluated using the multicenter arthroscopy hip outcome research network (MAHORN) classification with grade 4 or 5 defined as severe chondral lesions [32]. Chondral lesions of the femoral head were evaluated using the Outerbridge classification with grade 3 or 4 defined as severe chondral lesions [27].

### Surgical techniques

Supine hip arthroscopy under general and epidural anaesthesia was performed on a traction table with a well-padded peroneal post. An anterolateral portal (ALP), a mid-anterior portal (MAP) and a proximal mid-anterior portal (PMAP) were established. An interportal capsulotomy was used to improve arthroscopic visualisation and instrument navigation [23, 36]. The detached labrum was repaired with suture anchors if reparable. Microfracture was performed for severe (i.e., MAHORN grade 4 or 5) chondral defects. After releasing traction, cam osteochondroplasty was performed using a motorised round bur. Finally, capsular closure through the MAP and PMAP was performed with three–five stitches [36, 37].

### Rehabilitation

Patients were instructed to use flat-foot weight bearing for the first 2 weeks. If microfracture was performed during surgery, weight bearing limitations extended to 6 weeks. Patients were placed in a brace (Philippon hip brace; Bledsoe) for 2 weeks to protect the hip and limit flexion (0°–120°), abduction (0°–45°), and external rotation to 0°. Gentle passive ROM exercises were initiated during the first week under the supervision of a physical therapist. Circumduction at 70° of hip flexion and neutral hip flexion and continuous passive motion exercises were used to minimise adhesive capsulitis by applying 0°–90° of hip flexion for up to 4 h a day for 2 weeks.

Endurance strengthening commenced only after ROM was maximised and good stability in gait and movement was demonstrated. Patients were allowed progression of physical activity after passive symmetric pain-free ROM and normal gait pattern was achieved.

### Clinical outcomes

Total hip conversion, radiographic progression of osteoarthritis, and PROs were investigated. Patients completed comprehensive patient-reported outcome questionnaires to document outcomes preoperatively and at final follow-up. The questionnaires included the Non-Arthritic Hip Score (NAHS; out of 100 points) and the modified Harris Hip Score (MHHS; out of 100 points).

### Radiographic assessment

The radiographs of all patients were assessed using a picture archiving and communication system (PACS) with anteroposterior view of the pelvis and cross-table lateral and modified Dunn view (45° of hip flexion and 20° abduction in neutral rotation) [7]. LCE angle, Tönnis angle, and Sharp’s angle were measured in AP view with the patient in a supine position. All aforementioned measurements were rounded off to one decimal place. Shenton’s line was measured on a standing pelvic AP view, and the highest alpha angle was utilised for analysis of each hip [25] on cross-table lateral view or modified Dunn view [7]. LCE angle was formed by the intersection of a line drawn through the centre of the femoral head and extending to the lateral edge of the sourcil (the dense bone along the lateral edge of the weight-bearing region of the acetabulum), and a line perpendicular to one joining the two femoral head centres [38]. Tönnis angle was recorded as a measure of acetabular inclination, Sharp’s angle was used as a measure of acetabular index, and alpha angle was used as a measure of cam deformity.

### Intra- and interrater reliability of radiographic measurements

For intra-observer reliability, a single hip surgeon (blinded for review) measured each radiograph 3 times, with an interval of at least 1 week between measurements. For inter-observer reliability, 2 hip surgeons (EH and SU) who performed the radiograph review independently and were blinded to the clinical data and details of radiology reports measured for each radiograph. *K* values and intraclass correlation coefficient (ICC) of 1.0 are indicative of perfect agreement; the strength of agreement was interpreted as the following ICC values: 0.80 was almost perfect agreement, 0.61–0.80 was substantial agreement, 0.41–0.60 was moderate agreement, and 0.21–0.40 was fair agreement. Based on the standards for the *k* statistic proposed by Landis and Koch, there was substantial agreement between our measurements [18].

### Statistical analysis

Wilcoxon signed rank test and Kruskal–Wallis analysis were performed to compare preoperative and postoperative MHHS and NAHS among the three groups. Condition and chondral lesions among three groups were compared using Fisher’s exact test and post hoc Bonferroni correction. Statistical analysis was performed using SPSS software (version 24, SPSS Inc., Chicago, IL, USA). The level of statistical significance was set at *p* < 0.05.

An a priori power analysis was performed using G*Power (version 3.1, Universität Düsseldorf, Düsseldorf, Germany). According to previous reports, the THA conversion rate in those younger than 50 years old was considered 9%, and that in those older than 50 years old was 40% [14, 29]. Assuming Fisher’s exact test to analyse the 2 × 2 contingency table, a 2–1 group size ratio, and an alpha of 0.05, an odds ratio of 0.15 could be detected with 80% statistical power by including 72 samples. Considering the expected follow-up rate to be 90%, 80 patients were required.

## Results

### Patient characteristics

Patient characteristics are shown in Table 1. There were no significant differences of the distribution of FAI type among three groups. There were no statistically significant differences in gender, BMI, follow-up period, rate of sports participation, preoperative LCE angle, alpha angle, Sharp’s angle, or Broken Shentons’s line between groups. The median Tönnis angle was larger in the Middle age group, which was statistically significant.

**Table 1:** Demographic information

Characteristic	Younger age group	Middle age group	Advanced age group	*p* value
Number	57	18	9	
Age (year)	30.9 ± 10.9	56.7 ± 3.7	73.5 ± 2.5	
Male (%)	31 (54)	6 (33)	6 (67)	n.s.
Follow-up (month)	31.8 ± 13.5	30.9 ± 12.6	36.0 ± 15.8	n.s.
Sports participation (%)	41 (72)	9 (50)	5 (56)	n.s.
BMI	22.1 ± 3.1	22.5 ± 2.7	23.7 ± 2.9	n.s.
LCE angle	33.9 ± 6.2	35.1 ± 5.9	39.2 ± 7.6	n.s.
Sharp’s angle	41.1 ± 8.0	39.2 ± 3.3	37.0 ± 3.1	n.s.
Alpha angle	71.6 ± 13.2	67.4 ± 14.9	70.6 ± 11.8	n.s.
Tönnis angle	6.4 ± 4.5	8.7 ± 4.6	4.9 ± 5.7	0.04
Broken Shenton’s line	0	0	0	n.s.
FAI				
Cam type (%)	42 (74)	13 (72)	3 (33)	n.s.
Pincer type (%)	5 (9)	3 (17)	1 (11)	n.s.
Combined type (%)	10 (18)	2 (11)	5 (56)	n.s.

### Arthroscopic findings and procedures

The summary of arthroscopic findings and procedures is shown in Table 2. The rate of severe cartilage damage on the acetabular rim (MAHORN IV and V) was significantly higher in the Middle age group and in the Advanced age group compared to the Younger age group (*p* = 0.02 and 0.004, respectively). The prevalence of irreparable labrum in the Middle age group and in the Advanced age group was significantly higher than that in the Younger age group (*p* = 0.002 and 0.02, respectively). All labrums were treated with repair or reconstruction using iliotibial band autograft; labral debridement was not performed in any subjects.

**Table 2:** Arthroscopic hip findings and procedures

Findings and procedures	Younger age group	Middle age group	Advanced age group
Chondral lesion			
Acetabular rim			
MAHORN 0, I, II, III (%)	54 (95)	12 (67)	6 (67)
MAHORN IV, V (%)	3 (5)	6 (33)*	3 (33)*
Femoral head			
Outerbridge 0, I, II (%)	57 (100)	16 (89)	9 (100)
Outerbridge III, IV (%)	0 (0)	2 (11)	0 (0)
Labrum			
Repair (%)	55 (96)	14 (78)	5 (56)
Reconstruction (%)	2 (4)	4 (22)*	4 (44)*

*p < 0.05, compared with Younger age group

### Clinical outcomes

There were no major complications. Six patients in the Younger age group required revision arthroscopy due to cam lesion regrowth (one patient), residual cam lesion (one patient), progression of osteoarthritis (one patient) or postoperative adhesions (three patients). One patient in the Middle age group required revision arthroscopy due to postoperative adhesion. Three patients (17%) in the Middle age group underwent conversion THA. No patients underwent revision arthroscopy or conversion THA in the Advanced age group (Table 3).

**Table 3:** Postoperative clinical result

Characteristic	Younger age group	Middle age group	Advanced age group
Number	57	18	9
THA (%)	0 (0)	3 (17)	0 (0)
Additional surgery (%)	6 (11)	4 (22)	0 (0)

In all age groups, preoperative MHHS and NAHS significantly improved at final follow-up. There were no differences in preoperative MHHS (*p* = n.s.) but significant differences in preoperative NAHS among the three groups (*p* = 0.047). There were no significant differences in postoperative MHHS between the three groups (*p* = n.s.). In the Advanced age groups, postoperative NAHS was significantly lower than in the Younger age group (*p* = 0.003) (Table 4).

**Table 4:** Preoperative and postoperative modified Harris Hip Score (MHHS) and non-arthritic Hip Score (NAHS)

	Younger age group			Middle age group			Advanced age group		
	Pre-op.	Post-op.	*p* value	Pre-op.	Post-op.	*p* value	Pre-op.	Post-op.	*p* value
Median MHHS	69.3 (14.3–95.7)	100 (50.6–100)	0.001	60.0 (39.6–95.7)	95.7 (95.7–100)	< 0.001	61.6 (34.0–82.5)	98.4 (48.4–100)	0.004
Median NAHS	48.0 (11.0–75.0)*	69.3 (21.0–80.0)	< 0.001	35.5 (29.0–68.0)*	70.0 (59.0–80.0)	< 0.001	38.0 (27.0–51.0)*	61.0 (36.0–80.0)^†^	0.004

*p < 0.05, among the three groups

^†^*p* < 0.05, compare with the Younger age group

### Radiographic assessment

In the  Middle age group, the rate of progression to osteoarthritis was significantly higher than that in the Younger age group (*p* = 0.02). In the Younger age and the Middle age groups, preoperative Tönnis grade 1 had significantly higher rates of radiographic osteoarthritic progression than those with Tönnis grade 0 (Tönnis grade 1 vs 0, Younger age group, *p* = 0.02, Middle age group, *p* < 0.01) (Table 5).

**Table 5:** Preoperative and postoperative Tönnis classification grade

	Younger age group	Middle age group	Advanced age group
Pre-op. Tönnis			
Grade 0	45	12	4
Grade 1	12	6	5
Post-op. Tönnis			
Grade 0	44	11	4
Grade 1	10	2	5
Grade > 2	3	5	0
Progression of osteoarthritis (%)	4 (7)	6 (33)	0 (0)
Pre-op. Tönnis Grade 0 (%)	1 (2)	1 (5)	0 (0)
Pre-op. Tönnis Grade 1 (%)	3 (5)	5 (28)	0 (0)

Inter- and intra-observer reliability analyses of the radiographic measurements were assessed. The inter-observer/intra-observer ICCs of CE angle and Tönnis angle were 0.905/0.891 and 0.895/0.936, respectively. The inter-observer/intra-observer reliability of the alpha angle and Sharp angle were 0.729/0.769 and 0.718/0.841, suggesting a substantial agreement. Finally, measurements by a single observer were utilised for further analysis.

### Cox hazard model

The risk factors of either THA conversion or progression to osteoarthritis were investigated by Cox hazard modelling. In the Middle age group, preoperative Tönnis grade 1 radiographic osteoarthritis, and severe acetabular cartilage damage (MAHORN IV and V) were predictors of poorer outcomes (Table 6). Gender, BMI, sports participation, radiographic measurements, irreparable labrum and cartilage damage on the femoral head were not statistically significant risk factors.

**Table 6:** Risk factors for either THA conversion or radiographic OA progression

	Hazard ratio	95% CI	*p* value
Middle age group	6.62	1.87–23.8	0.004
Pre-op. Tönnis grade 1	3.29	1.51–7.14	0.003
MAHORN IV, V	2.63	1.41–4.90	0.002

## Discussion

The most important findings of the present study were significant improvement of PROs and no evidence of progressive osteoarthritis in patients aged over 70 years following hip arthroscopy for FAI. Preoperative PROs significantly improved postoperatively for FAI patients of all ages and the rates of irreparable labrum and severe chondral damage of the acetabular rim were significantly higher in patients aged over 50 years than that in patients aged younger than 50 years. The patients in their 50s and 60s had a higher risk of both THA conversion and progressive osteoarthritis. Significant predictors of poorer outcomes were age in 50s and 60s at the time of surgery, preoperative Tönnis grade 1 osteoarthritis, and severe acetabular cartilage damage observed at time of hip arthroscopy.

Hip arthroscopy is a useful tool for assessing and treating FAI, with many studies reporting favorable clinical outcomes after hip arthroscopy for FAI [2, 5, 23, 29]. In agreement with these studies, it was found that the median MHHS and NAHS significantly improved postoperatively in all age groups.

Some studies have shown that patients with osteoarthritis or severe cartilage damage at the time of surgery had worse clinical outcomes following arthroscopic FAI surgery. Mardones et al. reported that poor results were associated with Tönnis grade 2 osteoarthritis in patients aged over 60 years [21]. Philippon et al. showed that 20% of patients aged over 50 years who had ≤ 2 mm of joint space required conversion THA within 3 years of arthroscopy [30, 33]. Other authors indicated that the presence of osteoarthritic lesions on preoperative radiographs negatively affects clinical outcomes following hip arthroscopy [11, 13, 20]. Haviv et al. evaluated 535 patients (mean age 55 years) with osteoarthritis undergoing hip arthroscopic procedures including chondroplasty, debridement of teres ligament and/or acetabular labrum and reported that 90 (16%) patients required THA over a period of 7 years with predictors affecting time to THA being higher age and grade of osteoarthritis [13]. Gicquel et al. reported that Tönnis grade 1 hips had lower PROs and higher rate of osteoarthritis progression than Tönnis grade 0 hips [11]. Larson et al. evaluated 56 patients who had radiographic evidence of FAI with osteoarthritis (Tönnis grade more than 2) and reported that FAI correction in the presence of radiographic mild joint space narrowing resulted in improvement in pain and function at short-term follow-up [20]. Sansone et al. showed arthroscopic treatment for FAI with OA (Tönnis grade 1 or 2) resulted in improvement of postoperative PROs and 7% conversion THA rate [33]. In the current study, higher relative rates of osteoarthritic progression were observed in patients in their 50s and 60s. [15, 26, 30]. Preoperative Tönnis grade 1 was also associated with progression of radiographic osteoarthritis after hip arthroscopy. Cautious consideration and appropriate patient counselling are merited if planning hip arthroscopy for patients in their 50s and 60s with Tönnis grade 1 radiographic changes.

It is well known that cartilage damage is one of the most important predictors for affecting postoperative clinical outcomes. Therefore, although patients with Tönnis grade 2 or higher radiographic osteoarthritis were carefully excluded, 3 of 9 patients in their 70s and 6 of 18 patients in their 50s and 60s had severe chondral lesions (≥ MAHORN grade 4) of the acetabular rim. In a recent study, although patients had similar preoperative Tönnis grade at baseline, older patients had a higher rate of cartilage defects at the time of arthroscopy [24]. The findings of our study were similar to these previous findings. It is paramount to assess cartilage damage before surgery and render appropriate treatments at the time of surgery. Careful patient selection is required to reduce the rates of postoperative osteoarthritis progression in patients aged over 50 years.

Ben Tov et al. described improvement in clinical outcomes after arthroscopic labral repair for patients over 50 years old without radiologic signs of osteoarthritis [3]. The findings of our study were consistent with that study, demonstrating improvement in postoperative PROs despite some THA conversions in patients aged over 50 years [15, 26, 30]. Conversely, the rate of THA conversion in the oldest age group (i.e., 70s) was lower than that in other age groups. It is conceivable that, in general, older patients have lower activity levels, which may commensurately decrease symptoms requiring THA. Moreover, the lower postoperative NAHS in this oldest group of patients combined with the absence of conversion THA and radiographic arthritic progression may be consistent with the lower physical demands of these patients.

The study findings have significant clinical relevance. Chronological age in isolation is not an absolute contraindication to hip arthroscopy, relegating these patients to THA as their only surgical option. Select symptomatic patients even over 70 years of age that fail appropriate conservative treatment regimens with no or minimal arthritis (Tönnis 0 or 1) and without preoperative evidence of severe full thickness chondral lesions may be reasonable candidates for hip arthroscopy. As a minimally invasive procedure, hip arthroscopy may be an attractive surgical alternative to THA in this subset of elderly patients. Indeed, some patients deemed to have medical comorbidities precluding THA may be reasonable candidates for hip arthroscopy.

There were a number of limitations in this study. First, there was a small sample size in patients aged over 70 years old. However, our indication of surgery for older patients is only active patients without any osteoarthritis. Thus, it is difficult to collect data from a larger population. A further study with a larger population is merited. An a priori power analysis was performed using G*power 3 between the patients aged younger than 50 years and those over 50 years, but the results of this analysis were an effect size of *d* = 0.36 and exact power of 0.73. Therefore, we divided patients into three groups. Second, the data were limited to short-term follow-up. Further studies to investigate long-term follow-up clinical outcomes are necessary. Third, patients with bilateral symptomatic hip involvement were excluded, but this group may be a significant subgroup that deserves focused investigation. It was difficult to evaluate postoperative PROs with precision because the time interval between the first and second (contralateral) surgeries varied, and the MHHS and NAHS questionnaires included content to assess bilateral issues (e.g., walking). In fact, patients with bilateral involvement usually have weaker muscle strength and greater work disturbance than do patients with unilateral involvement. Fourth, this study was a retrospective study without a control group, such as patients who did not undergo surgery, and a randomised, controlled study with longer-term follow-up could provide valuable information. However, such a study may be difficult to perform. Fifth, recent studies revealed that the MHHS has a significant ceiling effect limiting its utility as an outcome measure in this patient population; however, the NAHS has less of a ceiling effect and was also used in this study. More clinically relevant PROs scores such as the International Hip Outcome Tool (iHOT-33) and Copenhagen Hip and Groin Outcome Score (HAGOS) may be helpful in these nonarthritic or minimally arthritic patient populations [17].

## Conclusion

Arthroscopic FAI correction and labral preservation surgery provides favorable clinical outcomes for patients aged over 70 years in the absence of significant osteoarthritis and severe acetabular chondral damage. Patients aged in their 50s and 60s have a higher risk of both THA conversion and progressive osteoarthritis, while patients aged over 70 years old show no evidence of progressive osteoarthritis. Chronologic age in isolation is not an absolute contra-indication for hip arthroscopy.
